# Effectiveness of ultrasound-guided dual nerve block in the below-knee amputation

**DOI:** 10.1186/s12893-023-02138-9

**Published:** 2023-08-10

**Authors:** Jung Wook Huh, Min Woo Kim, Young Min Noh, Han Eol Seo, Dong Ha Lee

**Affiliations:** Department of Orthopedic Surgery, Busan Medical Center, 62, Yangjeong-ro, Busan, Republic of Korea

**Keywords:** Diabetic foot, Amputation, Ultrasonography, Nerve block, Critical care, Postoperative care, Anesthesia, Local, Lower Extremity

## Abstract

**Purpose:**

Below knee amputation (BKA) is a common surgical procedure for diabetic foot ulcers and necrotizing lower limb fasciitis patients. However, it is a painful procedure and inadequate postoperative analgesia impedes rehabilitation and prolongs hospitalization. An ideal pain management regimen should provide superior analgesia while minimizing opioid consumption and improving rehabilitation.

**Methods:**

We retrospectively reviewed medical charts of 218 patients who underwent BKA for diabetic foot ulcer or necrotizing lower limb fasciitis at a single center between January 2017 and September 2020. Two groups were analyzed: patients who received dual nerve block (DNB) before surgery (Group I; *n* = 104), and patients who did not (Group II; *n* = 93). By the exclusion criteria, 21 patients were excluded. The femoral and sciatic nerves were each blocked separately under ultrasound guidance. This procedure was performed immediately before the operation.

**Results:**

Group I patients' subjective pain scores were significantly lower than that of Group II at 6, 12, and 24 h after BKA (*P* < 0.05). Group I’s morphine milligram equivalent (MME) was significantly lower than those of Group II at 72 h after BKA (*P* < 0.05). Moreover, the rate of postoperative nausea and vomiting (PONV) and delirium was significantly lower in Group I patients than that in Group II patients.

**Conclusion:**

Ultrasound-guided lower extremity nerve block surgery is excellent for early postoperative pain control, could be used as an accurate and effective pain control method, and can reduce the side effects of opioid consumption after BKA.

"A preprint has previously been published [1] "

## Introduction

The goal of lower extremity amputation is to maintain functional balance in the remaining lower extremities so that they can move independently. The most common cause of lower extremity amputation is peripheral vascular disease, and since it is particularly common in the elderly, medical conditions and socioeconomic conditions must be considered, so there are many difficult problems [2]. In addition, they must adapt to the use of prosthetic braces after amputation and undergo changes in their body image that are emotionally like those of losing a loved one. Emotional support and treatment after surgery are also important because changes in body image are associated with a happy mental and social life. In the case of lower extremity amputation, there are few reports on the methods of pain control after surgery. Intra-articular and peri-articular injections have been reported for effectively postoperative pain control up to 96 h after surgery, and continuous epidural injection has also been reported to have effective pain control, but various side effects such as hypotension and respiratory inhibition have been reported [3, 4]. There are restrictions on the use of analgesic medications in the patient group who underwent below-knee amputation (BKA) due to medical complications, and reports of effective pain control methods appear to be insignificant.

It is important to understand each site's surgical technique and anatomy for effective nerve block anesthesia [5]. When a long posterior flap technique is used for BKA, the common selection for amputation below the knee is 5.5 inches below the tibiofemoral joint line or 3.9–4.2 inches below the tuberosity of the tibia. As skin flaps may be accurately marked using a rule of thirds, the lower limb’s nerve innervations can be cleared [6]. Nerve innervation of the lower limb: the proximal anteromedial portion of the lower limb is innervated by the infrapatellar branch of the femoral nerve, and the medial portion of the lower limb is innervated by the saphenous branch of the femoral nerve, the proximal posterior portion of the lower limb is innervated by the posterior tibial nerve of the sciatic nerve, and the anterolateral portion of the lower limb is innervated by the common fibular nerve of the sciatic nerve. Accordingly, the method of effectively blocking the BKA region is a dual nerve block (DNB) method that blocks the sciatic nerve block and the femoral nerve block.

The ultrasound-guided block of the sciatic nerve and femoral nerve was performed by one experienced orthopedic surgeon that took less than ten minutes for each nerve block as a justification that this does not significantly add to the perioperative time. It also did not cause any local complications such as infection and hematoma at the injection site.

### Objectives of the study

In this study, the group that received DNB and the group that did not were divided into two groups with a time difference. From June 2018 to September 2020, all patients who underwent BKA had a dual nerve block. From January 2017 to May 2018, all patients who underwent BKA had no dual nerve block. The control group was controlled pain through intravenous self-regulatory pain treatment (IV PCA) and analgesic medication injection, while the subjects who performed US-guided DNB were set as the experimental group. This study aims to investigate the clinical benefits (pain control, minimizing opioid consumption, and enhancing rehabilitation) of US-guided DNB performed on the femoral sciatic nerves peripheral nerves that innervate the lower limbs—for patients undergoing BKA.

## Materials and methods

### Participants

We retrospectively reviewed medical charts of 218 patients who underwent BKA for diabetic foot ulcer or necrotizing lower limb fasciitis at a single center between January 2017 and September 2020. Disclosure of a potential conflict of interest -no financial support or benefits have been received by me, by any member of my immediate family, or any individual or entity in this study. Ethics approval was obtained from the Institutional Review Board "Public Institutional Review Board Designated by Ministry of Health and Welfare" (Approval # P01-202201-01-026). Informed consent for publishing this study is not applicable since it is a retrospective study.

**Fig. 1:**
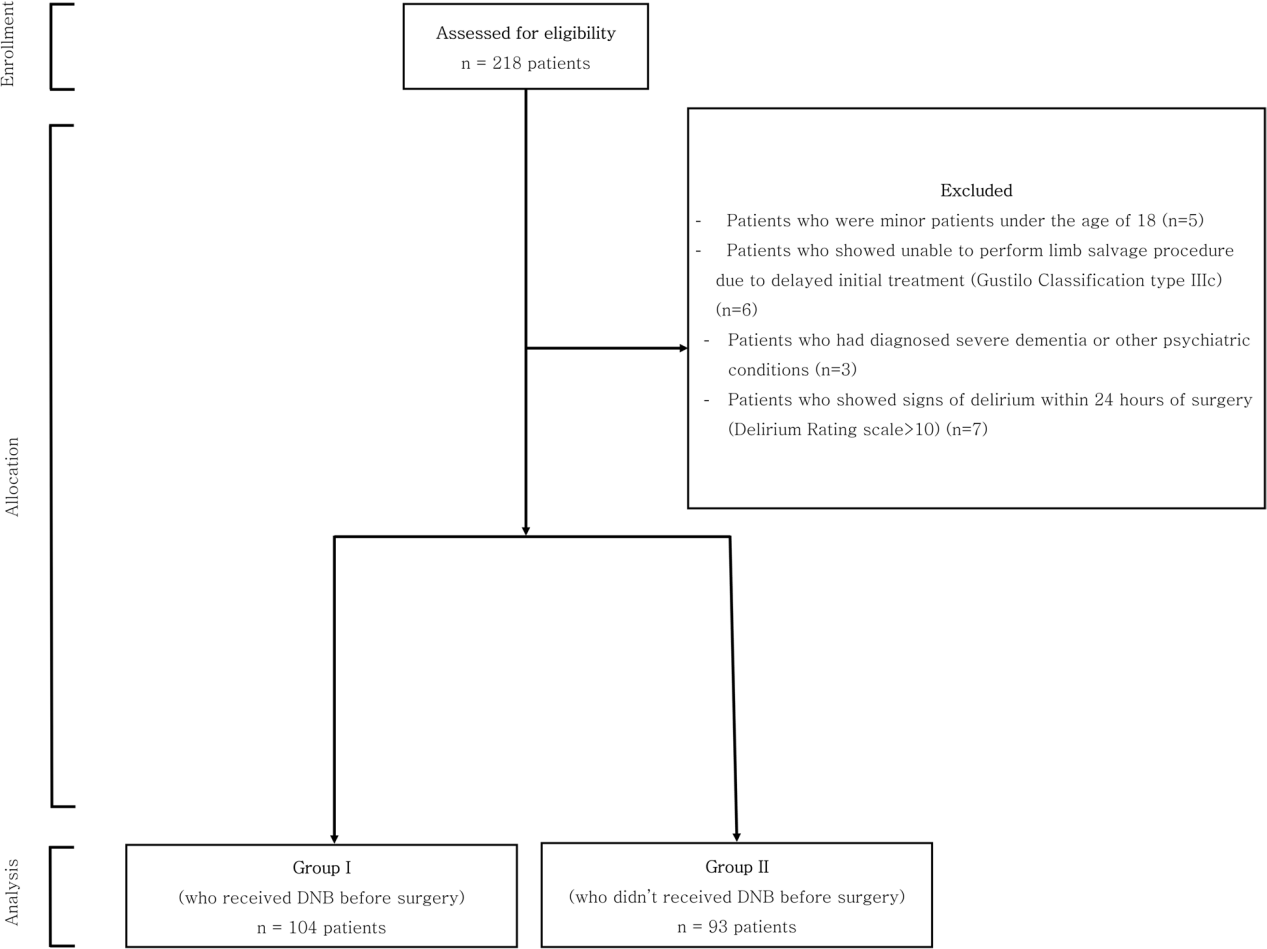
Flowchart for patient’s registration

Patients were excluded if they were minor patients under the age of 18 (*n* = 5), unable to perform limb salvage procedures due to delayed initial treatment (Gustilo Classification type IIIc) (*n* = 6), had history of dementia or other mental disorders (*n* = 3), or showed signs of delirium within 24 h of surgery (Delirium Rating scale > 10) (*n* = 7). By the above exclusion criteria, 21 patients were excluded from the study (Fig. 1).

### Trial design and study settings

All BKA was performed by a single orthopedic surgeon using a long posterior flap technique under general anesthesia [2]. The study design included 197 patients who underwent BKA, performed by a single orthopedic surgeon, and were divided into two groups: Group I, which received a dual nerve block (*n* = 104), and Group II, which did not receive a nerve block (*n* = 93). All patients underwent the same anesthesia procedures, and the same drugs were used across both groups to ensure standardization. All nerve blocks were performed after June 2018, over a period of 2 years and 3 months.

### Below-knee amputation (BKA) procedure

All patients were operated on by using the Burgess long posterior flap technique. The common selection for amputation below the knee is 5.5 inches below the tibiofemoral joint line or 3.9–4.2 inches below the tuberosity of the tibia. Mark the level for tibia cutting (A), determine a point that is about 2 cm distal for the anterior skin incision level and 15 cm distal for the posterior skin incision level, and draw a soft arc at each point to meet (A). An initial incision was made through the skin and subcutaneous fat with a scalpel and continued through the muscles of the anterior and fibular compartments with a diathermy blade. The vessels are identified before division and bound by absorbable suture material. The tibial nerve should be gently towed with a scalpel blade to identify and transpose the nerve vasa nervorum. Otherwise, it can cause troublesome bleeding deep in the wound. The fibula was removed, divided, and filled smoothly with the periosteum up to 2 cm above the skin incision. The tibia is also removed from the periosteum at a planned level of splitting and splits it with a hand or vibrating saw. The tibia is beveled and smoothed to prevent bone protrusion [2].

The soleus muscle should be excluded from the posterior sheath and cut horizontally in the bone section. The gastrocnemius muscle is thinned appropriately to cover the tip of the tibial bone. Excessive volume of the posterior flap may interfere with subsequent limb fitting aiming for a cylindrical stump. Before closure, attentive attention should be paid to the hemostasis and a drain inserted. The fascia is merged into sutures and closes the skin [2]. A long leg splint was applied for 2 weeks for preventing knee flexion contracture. Patients were also taught to change their lying positions and wore medical compression stockings on the contralateral side limbs throughout their hospital stay to help prevent postoperative complications. Postoperatively, physiotherapy begins with the goal of preventing contracture, limiting edema, and helping with general mobility in bed and moving. The amputee undergoes quadriceps muscle strengthening during this phase. Walking is initially resumed gradually with the assistance of walking aids.

### Interventions

**Fig. 2:**
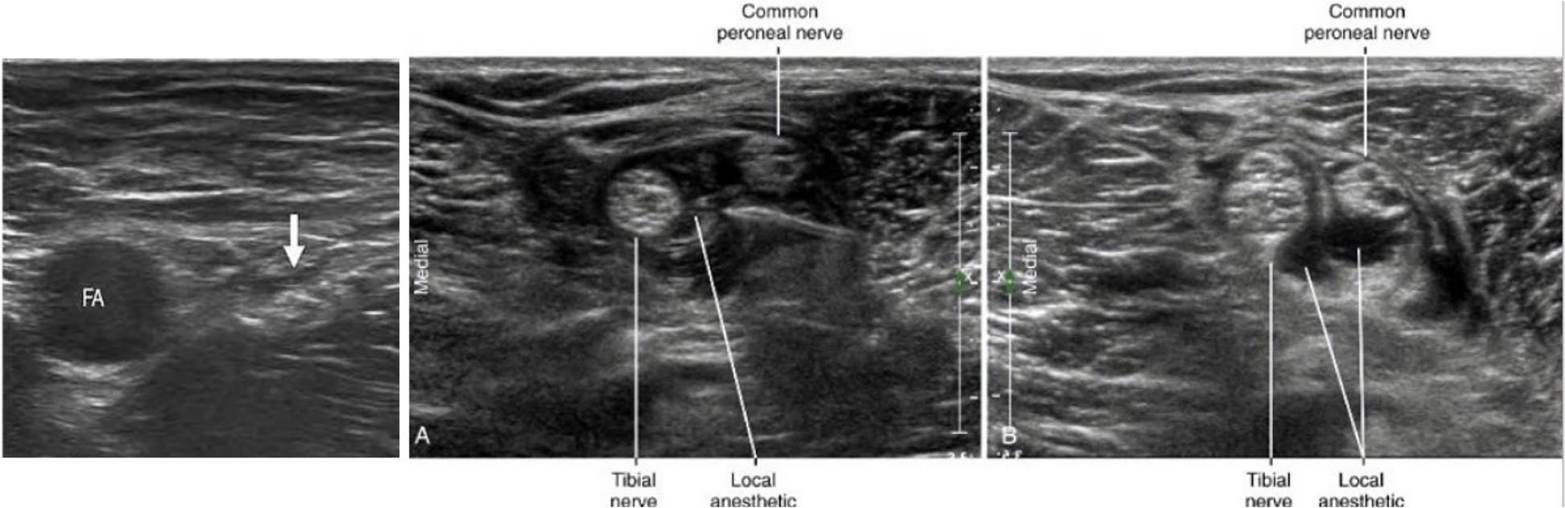
Dual Nerve block. **A** Femoral nerve block using ultrasonography; Place the needle near the femoral nerve(arrow). Femoral artery (FA). **B** Posterior tibial nerve Common fibular nerve block using ultrasonography; Place the needle near the posterior tibial nerve and common fibular nerve. **C** After local anesthetics injection

For Group I, were performed by a single experienced orthopedic surgeon immediately before surgery. All nerve blocks were performed using a 5-cm long, 5-12 MHz linear probe (LOGIQ e, GE, Boston, USA) and a 22G spinal needle. Under US guidance, the femoral (3-in-1 block) [3] and sciatic nerve (posterior popliteal approach) [4] were each blocked using 7.5 and 8 ml respectively, of 0.75% ropivacaine mixed with 7.5, 8 ml of 1% lidocaine at a 1:1 ratio (Fig. 2) [7]. A sensory test including a pinprick test was done after performing the block to confirm a successful block. Patients were given 200 mg of Celebrex (celecoxib; Pfizer, NY, USA) as premedication. During the operation, no additional local anesthetic or analgesic medication was administered intra-operatively. Group II did not receive the nerve block but underwent the same general anesthesia procedure as Group I.

For postoperative pain management, both groups received the same regimen. A patient-controlled intravenous analgesia mix was used, which included 2 mg butorphanol (butorphanol tartrate; Myungmoon Pharm., Seoul, Korea), 50 mg tridol (tramadol hydrochloride; Yuan Corp., Seoul, Korea), and 30 mg ketorak (ketorolac tromethamine; Hanmi Pharm., Seoul, Korea) in 100 mL of saline (background infusion rate 0.05 ml/hr, bolus 1 ml, lockout 10 min). It’s calculated as 19 MME (morphine milligram equivalent). The number of administrations was confirmed by checking the remaining dose on the 3rd day after surgery. Additionally, a rescue analgesic (50 mg of Tramadol) (5 MME), was provided for both groups for additional postoperative pain control.

By maintaining the same anesthesia procedures, drugs, and postoperative pain management strategies across both groups, we aim to isolate the effect of the dual nerve block on postoperative outcomes.

#### Outcome assessments

Post-operative pain intensity was measured in the electronic medical records by visual analog scale (VAS) at 6, 12, 24, 48, 72 h after surgery. Before the initial measurement of VAS, medical staff gave a detailed explanation of the meaning of VAS. Several doses administered by PCA were recorded for the first 72 h. In addition, the number of rescue analgesics was calculated as MME. Notes on Mangled Extremity Severity Score (limb salvage procedure index 0–11) and American Society of Anesthesiologists (ASA Grade I-V) were compared. Furthermore, out-patient department 6 months follow-up complications (Neuroma formation, Osteomyelitis, Bony erosion, Ulceration, and ongoing ischemia), local complications (Stump hematoma, Flap necrosis or infection, Stump trauma from falls, and Nerve block site hematoma or infection), general postoperative complications (Pressure sore, Pneumonia, Deep Vein Thrombosis, Postoperative nausea and vomiting, Delirium) and length of hospitalization were compared.

#### Statistical analysis

Statistical analyses were performed using IBM SPSS ver. 22.0 software (IBM Corp., Armonk, NY, USA). The paired t-test was used to verify differences in clinical results and patient demographics between the two groups. Power analysis revealed an effect size of 0.5, a statistical significance of 0.05, and statistical power of 0.91 for both groups.

## Results

There were no significant differences in age (Group I: 62.56 ± 8.22, Group II: 63,12 ± 7.58), gender, sex, BMI (kg/m2), Mangled Extremity Severity Score (MESS), VAS measured in the ward on the day of admission, American Society of Anesthesiologist (ASA), operation time, blood loss, urine output (Table 1). Group I patients' subjective pain scores were significantly lower than those of Group II at 6, 12, and 24 h after BKA (*P* < 0.001, *P* < 0.001, *P* = 0.025). Post-operative VAS scores at 48 and 72 h did not show statistically significant differences (Table 2).

**Table 1:** Patient demographics

	**I**^**d**^**(*n* = 104)**	**II**^**e**^**(*n* = 93)**	***p*-value**
**Mean Age (years)**	62.56(8.22)	63,12(7.58)	0.33
**Gender**			0.16
**Male**	78	74	
**Female**	26	19	
**Mean BMI**^**a**^**(kg/m**^**2**^**)**	22.08(1.51)	22.05(1.42)	0.48
**Mean MESS**^**b**^	7.21(0.82)	7.45(1.13)	0.26
**Mean VAS measured in ward on the day of admission**			
	5.76(1.92)	5.87(1.83)	0.34
**ASA **^**c**^** (Grade)**			
**I**			0.16
**II**	2	1	
**III**	28	23	
**IV**	45	41	
**V**	24	22	
	5	6	
**Mean**	91.57(11.02)	96.15(12.78)	0.06
**Operation time (min)**			
**Mean Blood loss (ml)**	243(18.46)	269(20.17)	0.13
**Mean Urine output (ml)**	325.02(21.92)	290.67(19.81)	0.07

^a^*BMI* Body mass index

^b^*MESS* Mangled Extremity Severity Score

^C^*ASA* American Society of Anesthesiologist

^d^I: Dual Nerve Block Group

^e^II: Control group(No injection)

**Table 2:** VAS* for post-operative (mean, standard deviation) and PCA* consumption for post-operative 72 h (mean, standard deviation)

	**I**^**‡**^**(*n* = 104)**	**II**^**§**^**(*n* = 93)**	***p*-value**
6 h	1.71(0.41)	7.28(2.50)	*P* < 0.00*
12 h	2.81(0.72)	6.29(1.85)	*P* < 0.00*
24 h	4.53(1.15)	5.61(1.52)	0.03*
48 h	3.45(1.08)	3.82(0.92)	0.32
72 h	3.52(0.55)	3.62(0.71)	0.28
PCA* consumption (ml)	75.72(9.78)	87.90(17.39)	*P* < 0.00*
MME*	18.60(3.72)	21.11(4.88)	*P* < 0.00*
Additional analgesic injection (number of injection)	1.25(0.32)	1.88(0.65)	*P* < 0.00*

^*^VAS: Visual analog scale at

Hours Post-op: Hours of the post-operative

‡I: Dual Nerve Block Group

§II: Control group(No injection)

*Significant difference between Group I and Group II

*PCA: patient controlled analgesia

* MME: morphine milligram equivalent

Group I used a smaller volume of PCA solution (75.72 ± 9.78 mL) in the first 72 h after surgery compared to Group II (87.90 ± 17.39 mL). Morphine milligram equivalent (MME) was 18.60 ± 3.72 for Group I patients and 21.11 ± 4.88 for Group II patients. Several injections administered by PCA were significantly lower for Group I as well, with an average of 1.25 ± 0.32 injections for Group I patients and 1.88 ± 0.65 injections for Group II patients. (Table 2).

There were no statistically significant differences in the incidence of pressure sores or pneumonia between the groups. However, Group I’s incidence of postoperative nausea and vomiting (PONV) (0.00%) and delirium (0.05%) was lower than Group II, respectively (0.15%), (0.21%). There were also no reports of any local complications due to DNB, nor any reports of block failure. There were no statistically significant differences in local complications (Stump hematoma, flap necrosis or infection, stump trauma from falls) or length of hospital stay between the groups. There were also no statistical differences in out-patient department 6 months follow-up complications (Neuroma formation, Osteomyelitis, Bony erosion, Ulceration, and ongoing ischemia). (Table 3).

**Table 3:** Complications & length of stay

	I^*^(*n* = 104)	**II**^**†**^**(*n* = 93)**	***p*-value**
General Complications			
Pressure sore	**3**	**2**	**0.86**
Pneumonia	**2**	**1**	**0.48**
DVT^‡^	**0**	**0**	**-**
Postoperative nausea and vomiting	**1**	**11**	0.02*
Delirium	**2**	**9**	0.02*
Local Complications	**2**	**2**	**0.90**
Stump hematoma	**3**	**2**	**0.78**
Flap necrosis or infection	**1**	**1**	**0.96**
Stump trauma from falls	**0**	**-**	**-**
Nerve block site hematoma or infection			
Length of stay (days) (mean)	**26.57(5.12)**	**29.14(6.88)**	**0.09**
OPD” 6 months follow-up complications			
Neuroma formation	**1**	**0**	**0.45**
Osteomyelitis	**2**	**2**	**0.90**
Bony erosion	**0**	**1**	**0.42**
Ulceration and ongoing ischemia	**2**	**2**	**0.90**

^*^Dual Nerve Block Group

†II Control group(No injection)

‡DVT: Deep vein thrombosis,”Out-patients department

*Significant difference between Group I and Group II

## Discussion

The combined femoral distal sciatic nerve blocks provided reliable postoperative pain control and effectively reduced opioid consumption for BKA. DNB can achieve adequate pain control in this study up to 24 h after surgery. Besides pain control, general complications such as the incidence of PONV and delirium were remarkably reduced due to the DNB effect. Its results are quite different from other studies in that there was no significant difference in delirium rate in CFNC (continuous femoral nerve catheter) group and SA (standard analgesia) group [5]. Considering the etiology of delirium is complex and multifactorial, it is remarkable that controlling pain adequately can be a powerful management of delirium.

Several advantages of perioperative nerve block techniques were reported besides our study. Based on the work by Farrar et al., with the utilization of a continuous femoral nerve catheter, the 60% pain score was reduced preoperatively, as well as 50% and 54% lower pain scores on postoperative days 1 and 2, respectively [6]. According to Rongguo Yu et al., patients who received CACB (continuous adductor canal block) had better VAS scores with rest up to 48 h postoperative mobilization 48 h, and rescue analgesia than those that underwent SACB (single adductor canal block) [8]. Considering that DNB has only a 24-h effect, the comparison of pain control with continuous femoral nerve catheter dwelling or sciatic nerve catheter dwelling may be meaningful in further study.

Our study found that a previous study found that continuous femoral nerve block reduced the patient-reported mean pain score, morphine equivalent consumption, as well as lower rates of opioid-related adverse events during the perioperative period [5]. This is consistent with the findings that controlling pain and reducing opioid requirements are associated, with reduces PONV and delirium, and adds to the body of evidence that regional techniques can improve postoperative complications.

Considering that many elderly patients have degraded system function; cardiovascular function, especially lower extremity nerve blocking, is a relatively safe technology [9–11]. It does not significantly impair cardiac or respiratory function (except for lidocaine toxicity or epidural diffusion).

One of the major side effects of the femoral nerve block has been addressed in previous studies [5, 12]—cause temporary weakness in the quadriceps muscles. There were concerns about whether these side effects delay the patient's start of rehabilitation by continuously performing nerve block in this study. However, patients undergoing BKA surgery were instructed not to come down from the bed due to the risk of falling for about 72 h and crutch walking for 6 to 12 weeks has been recommended [13–16]. Therefore, it was not a problem regardless of the duration of the side effects of the nerve block.

Several factors, including the presence of an amputation site or pre-amputation pain, are positively correlated with the onset of phantom limb pain [17]. The latter can often be treated successfully through discussion of combinations of amitriptyline and gabapentin or pregabalin as the primary drug therapy for phantom limb pain. The use of metallic stump liners (Farabloc™) may also attenuate phantom pain. However, studies on postoperative pain management through nerve block are insufficient. Although there is no evidence that epidural anesthesia can reduce phantom pain during surgery, it has a good analgesic effect before and after surgery [2]. Seeing that amputation pain has a positive correlation with phantom limb pain, it is expected that perioperative amputation pain management can reduce phantom limb pain.

The limitations of our study must be acknowledged. Random double-blind studies may be needed to confirm these results. We also did not consider the number of narcotics and antiemetics before and during surgery that could affect postoperative pain and drug-related side effects. Since the number of registered patients is limited, further research may be needed for more accurate results. Patients who had an overall follow-up rate of > 70% at 6 months but failed to follow-up (including 25 patients without follow-up data) had a lower socioeconomic status than those with complete follow-up. Therefore, the outcome may underestimate the overall degree of disability. However, follow-up loss rates were similar in both groups, and all available data from 159 people who received at least one follow-up evaluation were used for analysis.

## Conclusions

Ultrasound-guided lower extremity nerve block surgery is excellent for early postoperative pain control, could be used as an accurate and effective pain control method, and can reduce the side effects of opioid consumption after BKA.

### What’s known/what’s new statements

Below-knee amputation (BKA) is a painful procedure. Ultrasound-guided dual nerve block (DNB) can be an adequate painkiller by performed on the femoral, and sciatic nerves for undergoing BKA. The DNB group is excellent for early postoperative pain relief and can reduce the side effects of opioid consumption.

## Data Availability

The datasets generated during and/or analyzed during the current study are not publicly available due to the IRB ("Public Institutional Review Board Designated by Ministry of Health and Welfare") guideline, it is stipulated that the patient’s personal data should be discarded within 6 months after data collection.
